# Parietal Perfusion Alterations in Parkinson's Disease Patients Without Dementia

**DOI:** 10.3389/fneur.2020.00562

**Published:** 2020-06-23

**Authors:** Laura Pelizzari, Sonia Di Tella, Federica Rossetto, Maria Marcella Laganà, Niels Bergsland, Alice Pirastru, Mario Meloni, Raffaello Nemni, Francesca Baglio

**Affiliations:** ^1^IRCCS, Fondazione Don Carlo Gnocchi, Milan, Italy; ^2^Buffalo Neuroimaging Analysis Center, Department of Neurology, Jacobs School of Medicine and Biomedical Sciences, University at Buffalo, State University of New York, Buffalo, NY, United States; ^3^Department of Pathophysiology and Transplantation, Università degli Studi di Milano, Milan, Italy

**Keywords:** arterial spin labeling, cerebral blood flow, gray matter, cognitive decline, Parkinson's disease, magnetic resonance imaging

## Abstract

Fronto-parietal regions are involved in cognitive processes that are commonly affected in Parkinson's disease (PD). The aims of this study were to investigate cerebral blood flow (CBF) and gray matter (GM) volume within the regions belonging to the fronto-parietal circuit in people with PD (pwPD) without dementia, and to assess their association with cognitive performance. Twenty-seven pwPD without dementia (mean [SD] age = 67.4 [8.1] years, 20 males, mean [SD] Montreal Cognitive Assessment, MoCA score = 24.2 [2.9], median [IQR] Hoehn and Yahr scale = 1.5 [1–2]) and twenty-six age- and sex-matched healthy controls (HC) were scanned with arterial spin labeling (ASL) and T1-weighted magnetic resonance imaging (MRI) sequences to investigate CBF and GM volume, respectively. The cognitive performance of the enrolled pwPD was assessed with MoCA, Trail Making Test (TMT, part A, B, B-A), phonemic fluency and semantic fluency tests. The scores were adjusted for age and education. After standard preprocessing, CBF differences between pwPD and HC were tested with a voxel-wise approach. Voxel-based morphometry was used to compare pwPD and HC in terms of GM volume. Both voxel-wise comparisons between pwPD and HC were restricted to regions of the fronto-parietal circuit. The following additional voxel-wise analyses were performed within regions showing either perfusion or GM volume alterations: (1) correlation with neuropsychological test scores; (2) subgroup comparison after median split on each neuropsychological test score. Family-wise error-corrected (FWE) *p*-values lower than 0.05 were considered significant. Significant hypoperfusion was identified in the left inferior parietal lobule (IPL, p_peak_ = 0.037) and in the bilateral superior parietal lobule (SPL, left hemisphere: p_peak_ = 0.037; right hemisphere: p_peak_ = 0.049) of pwPD when compared to HC. No significant GM atrophy was observed. Local hypoperfusion did not correlate with any neuropsychological test scores. However, significantly lower CBF was observed in the left SPL and IPL of the pwPD subgroup who performed poorer on TMT part A in comparison with the pwPD subgroup that performed better. Perfusion alterations may occur in parietal regions of pwPD without dementia, and may be associated with lower visuomotor skills. Parietal CBF may be considered as a suitable early biomarker for longitudinal studies investigating cognitive decline in PD.

## Introduction

Parkinson's disease (PD) is a neurological disorder that is prominently characterized by motor symptoms, including bradykinesia, rigidity, resting tremor and gait disturbance (1). Besides these hallmarks, cognitive deficits have been identified as an important non-motor manifestation of the disease (2). Cognitive decline typically occurs gradually, after a period of pure motor symptoms (3). Specifically, executive dysfunction may occur in the milder stages of PD, while global dementia can develop as the disease progresses (4).

Alterations in the neural circuits including subcortical structures and frontal and parietal cortices are thought to be associated with PD cognitive deficits (5). Two profiles of neuropsychological deficit were proposed in PD: working memory and executive function deficits, reflecting fronto-striatal dysfunction, and visuospatial function and semantic fluency impairment, indicative of posterior cortical dysfunction (6). Reduced dopamine uptake in the caudate was observed in persons with PD (pwPD) presenting with executive dysfunctions (7), and bilateral caudate involvement was associated with increased risk of developing cognitive impairment (8). In addition, lower functional connectivity in the fronto-parietal network was reported to be associated with mild cognitive impairment (MCI) in pwPD (9). Significantly reduced regional cerebral blood flow (rCBF) in frontal and parietal cortices was detected with single photon emission computed tomography (SPECT) in demented pwPD (10). Furthermore, fluorodeoxyglucose (FDG) positron-emission tomography (PET) studies showed an association between reduced metabolism in prefrontal and parietal cortex and cognitive impairment in PD (11), and a declining metabolism in these brain areas as the disease progresses (12). Besides metabolic deficits, brain atrophy is the most commonly documented imaging correlate of PD with established dementia (13). Gray matter (GM) volume loss in cognitively impaired pwPD was observed in widespread cortical and subcortical structures involved in cognitive functions (3).

Despite the consistent evidence of structural, functional, and metabolic alterations throughout the brain in demented pwPD, it remains unclear whether some changes are present even in absence of frank cognitive deficits and whether some imaging biomarkers can predict cognitive decline in PD. Several studies reported no cortical structural alterations in cognitively preserved pwPD, making GM volume loss less obvious as a prodromal biomarker for cognitive decline in PD. Nevertheless, FDG cortical hypometabolism was reported even in non-demented pwPD, and parietal metabolism was suggested as a risk factor for cognitive decline (14, 15). In addition, arterial spin labeling (ASL) magnetic resonance imaging (MRI) revealed reduced perfusion in a group of pwPD without dementia (16), and Montreal Cognitive Assessment (MoCA) score was reported as a significant predictor of ASL-derived hypoperfusion in posterior parieto-occipital regions, middle, and superior frontal gyri dorsolateral prefrontal cortex and pre- and post-central gyri in pwPD (17).

ASL is an MRI technique that quantitatively assesses brain perfusion in terms of cerebral blood flow (CBF). Due to the neurovascular coupling, CBF varies in proportion to the local energy consumption and the metabolic needs of the brain (18). For this reason, ASL was suggested as a proxy technique to investigate the patterns of metabolic alterations (19). Compared to FDG PET, ASL has the advantage of not requiring any injection of radiotracers or exogenous contrast agents, making its acquisition more acceptable (20). ASL studies have found evidence of hypoperfusion patterns that mirror those seen with FDG PET in Alzheimer's disease and fronto-temporal dementia (19, 21, 22). Conversely, patterns of ASL-derived hypoperfusion have not yet been confirmed as a valuable biomarker for the risk of developing cognitive impairment in PD.

Our objective was first to assess CBF within the caudate nucleus and fronto-parietal brain regions in a group of non-demented pwPD using ASL MRI, to test whether perfusion is altered in these areas in PD in absence of frank cognitive impairment. Second, we aimed to evaluate GM volume in the same regions and to assess if hypoperfusion is also reflected by atrophy. Finally, this study aimed to test if either CBF or GM volume alterations are associated with the cognitive performance, to probe ASL and structural MRI as possible prodromal biomarkers for cognitive decline in PD.

## Materials and Methods

### Participants

Fifty-three subjects [27 pwPD and 26 healthy controls (HC)] participated in this study. PwPD were recruited from the Neurorehabilitation Unit of the IRCCS Fondazione don Carlo Gnocchi in Milan, Italy, while most of HC were volunteers and personnel from our Institute. Being left-handed or presenting with neurological diseases other than PD, psychiatric disorders, cardiovascular and/or metabolic diseases were considered as exclusion criteria for this study. Inclusion criteria for the pwPD participating in the study were: (1) diagnosis of probable PD, according to the Movement Disorder Society (MDS) Clinical Diagnostic Criteria for PD; (23) (2) positive DaT scan; 3) mild to moderate stages of the disease (Modified Hoehn and Yahr-H&Y <3); (23) (4) time spent with dyskinesias assessed with the MDS-sponsored revision of the Unified Parkinson's Disease Rating Scale (MDS-UPDRS), part IV lower than 2; (23) (5) stable dopaminergic therapy for at least 3 months; 6) MoCA score adjusted according to Santangelo et al. (24) higher than 15.5.

All the enrolled pwPD underwent a clinical evaluation by an experienced neurologist, who assessed the severity of motor symptoms with the H&Y Scale (25) and the MDS-UPDRS part III (23). The global cognitive performances of pwPD who participated in the study were assessed by experienced neuropsychologists. Specifically, the cognitive assessment included MoCA, Trail Making Test (TMT, part A, B, B-A), phonemic fluency and semantic fluency tests. The scores of neuropsychological tests were adjusted for age and education (24, 26–28). The levodopa equivalent daily dose (LEDD) was computed for all the pwPD (29).

Body mass index (BMI) was recorded for all the participants.

The study was approved by the Ethics Committee of IRCCS Fondazione don Carlo Gnocchi and it was performed in accordance with the principles of the Helsinki Declaration. Written and informed consent was provided by all the participants.

### MRI Acquisition

All scans were acquired on the same 1.5T Siemens Magnetom Avanto scanner, with a 12-channel head coil. Each participant was scanned according to the following MRI protocol: (1) dual-echo turbo spin echo proton density PD/T2-weighted sequence [repetition time (TR) = 5,550 ms, echo time (TE) = 23/103 ms, matrix size = 320 × 320 × 45, resolution 0.8 × 0.8 × 3 mm^3^]; (2) 3D high-resolution magnetization-prepared rapid acquisition with gradient echo (MPRAGE) sequence (TR = 1,900 ms, TE = 3.37 ms, TI = 1,100 ms, matrix size = 192 × 256 × 176, resolution 1 × 1 × 1 mm^3^); (3) 2D T1-weighted sequence (TR/TE = 393/12 ms, matrix size = 128 × 128 × 26, resolution = 1.7 × 1.7 × 5 mm^3^); (4) multi-delay pseudo-continuous ASL (pCASL) sequence with background suppressed 3D gradient and spin echo (GRASE) readout (30) (TR/TE = 3500/22.58 ms, labeling duration = 1500 ms, 5 post-labeling delays (PLD) = [700, 1200, 1700, 2200, 2700] ms, 12 pairs of tag/control images for each delay, matrix size = 64 × 64 × 32, resolution = 3.5 × 3.5 × 5 mm^3^, distance between the center of imaging slices and labeling plane of 90 mm; 3 M0 images acquired with TR = 5,000 ms).

### MRI Processing

All the MRI processing was performed with FMRIB's Software Library (FSL, http://www.fmrib.ox.ac.uk/fsl), unless otherwise specified.

White matter (WM) T2-hyperintensities were segmented on the PD/T2-weighted images by an experienced operator with Jim 6.0 software package (http://www.xinapse.com/). The T2-hyperintensity masks were registered to the respective MPRAGE T1-weighted image with Advanced Normalization Tools (ANTs—http://stnava.github.io/ANTs). The registered masks were thresholded at 0.5 and binarized.

N3 bias field correction was applied to MPRAGE T1-weighted images, then lesion filling tool was used to correct for WM T1-hypointensities concurrent to the T2-hyperintensities (31). Brain extraction was performed (32) and SIENAX software tool was used to derive WM, GM, and cerebrospinal fluid (CSF) masks for each subject (33).

Partial volume GM maps were non-linearly registered to Montreal Neurological Institute (MNI) standard space with ANTs (http://stnava.github.io/ANTs). The Jacobian determinant image of the transformation was derived with ANTs (http://stnava.github.io/ANTs). Each registered GM map was multiplied by the respective Jacobian determinant image, and then smoothed with a Gaussian kernel (sigma = 3 mm).

ASL data were realigned with ANTs (http://stnava.github.io/ANTs) to correct for movements. Once realigned, the 12 pairs of tag/control images for each delay were averaged, and the tag images were subtracted by the respective control ones. The obtained perfusion-weighted images were used to compute the CBF map with oxford_asl tool (34) (tissue T1 = 1.2 s, T1 of blood = 1.36 s, tagging efficiency = 0.8) (30, 35). CSF magnetization was estimated from the M0 image and CBF maps were calibrated accordingly, with asl_calib tool (34). Partial volume effect (PVE) correction was performed, assuming a perfusion ratio between GM and WM of 2.5, as described in Marshall et al. (36) and Pelizzari et al. (37). GM CBF maps were registered to MNI standard space with ANTs (http://stnava.github.io/ANTs), by applying a concatenation of transformations, as follows: (1) linear, from ASL space to the respective 2D T1-weighted image (presenting with the same slice thickness of ASL data); (2) linear, from 2D T1-weighted image to the respective MPRAGE; (3) non-linear, from MPRAGE to MNI standard space.

### Statistical Analysis

The assumption of normality of data distribution was tested with Shapiro-Wilk test. Parametric or non-parametric statistics were used, as appropriate. Demographic characteristics were compared between pwPD and HC groups.

Voxel-wise comparisons between pwPD and HC in terms of both CBF and GM volume were performed with randomize tool (5,000 permutations, cluster detection with threshold-free cluster enhancement) (38). Age was included as a covariate in both the general linear models (GLM). Given that the Jacobian determinants derived with ANTs include the linear registration component, voxel-based morphometry (VBM) analyses were controlled also for the scaling factor derived with SIENAX (33). Both CBF and GM volume voxel-wise comparisons between pwPD and HC were restricted to specific ROIs. The region of interest (ROI) mask used for this study included the caudate nucleus [defined according to the Harvard-Oxford subcortical structural atlas (39)], Brodmann area (BA) 9, BA 10, and BA 46 [defined according to the Sallet's dorsal frontal parcellation atlas (40), thr = 50%], superior parietal lobule (SPL) regions [defined and labeled according to the Mars' parietal cortex atlas (41), thr = 75%] and inferior parietal lobule (IPL) regions [defined and labeled according to the Mars' parietal cortex atlas (41), thr = 75%].

If either perfusion or GM volume alterations were detected in pwPD with respect to HC, voxel-wise correlations between neuropsychological test scores (NPS) and either CBF or GM volume were assessed in pwPD with randomize tool (5,000 permutations, threshold-free cluster enhancement for cluster detection) (38), to assess if the observed MRI alterations were mirrored by the cognitive performance. NPS-CBF and NPS-GM volume voxel-wise correlations were restricted to the regions showing either perfusion or GM volume alterations, respectively. A specific GLM was defined for each neuropsychological test (i.e., MoCA, TMT part A, TMT part B, TMT part B-A, phonemic fluency and semantic fluency); scores adjusted for age and education were used.

In order to further assess the association between cognitive performance and either CBF or GM volume alterations, pwPD were split into two subgroups according to the median score at each neuropsychological test (i.e., median split in the pwPD group). CBF within areas of hypoperfusion and GM volume within atrophic regions, if any, were compared between each pair of pwPD subgroups with randomize tool (5,000 permutations, cluster detection with threshold-free cluster enhancement) (38).

The results of all the voxel-wise analyses were Family-Wise Error (FWE) corrected to account for multiple comparisons (38, 42). FWE-corrected *p*-values lower that 0.05 were considered significant. Significant clusters in the cortex were mapped according to Sallet's dorsal frontal parcellation atlas (40) and Mars's parietal cortex atlas (41).

## Results

### Demographic and Clinical Characteristics of the Participants

Demographic and clinical information of pwPD and HC groups are reported in Table 1. The groups were age-matched (mean age [SD] = 67.4 [8.1] years vs. 66.1 [7.5] years for pwPD and HC respectively, *p* = 0.527) and sex-matched (20 males vs. 17 males in pwPD and HC groups respectively, *p* = 0.491). Although the pwPD group presented with a mean BMI within the overweight range, no differences in terms of BMI were found between pwPD and HC groups (mean BMI [SD] = 25.3 [2.6] vs. 24.2 [3.6], in pwPD and HC respectively, *p* = 0.118). The recruited pwPD presented with a median (IQR) H&Y of 1.5 (1–2) and mean (SD) MDS-UPDRS III score of 19.7 (12.1). The score from the neuropsychological examination of pwPD, adjusted for age and education, are shown in Table 2. The pwPD had a mean (SD) MoCA score adjusted for age and education of 24.2 (2.9). For the TMT, the pwPD performed as follows: median (IQR) TMT A score of 42 (30–56), median (IQR) TMT B score of 73 (58–139), and median (IQR) TMT B-A score of 40 (18–68). The pwPD mean (SD) phonemic and semantic fluency scores at were 35.4 (9.5) and 42.9 (8.5), respectively.

**Table 1:** Demographic and clinical characteristics of the recruited pwPD and HC groups.

	**pwPD (*n* = 27)**	**HC (*n* = 26)**	**pwPD vs. HC *p*-value**
Males, *n* (%)	20 (74)	17 (65)	0.491^a^
Age in yrs, mean (SD)	67.4 (8.1)	66.1 (7.5)	0.527^b^
BMI, mean (SD)	25.3 (2.6)	24.2 (3.6)	0.118^b^
HandY, median (IQR)	1.5 (1–2)	-	-
MDS-UPDRS III, mean (SD)	19.7 (12.1)	-	-
Disease duration in yrs, median (IQR)	3 (2–5)	-	-
Clinical onset laterality, left *n* (%)	12 (44.4)	-	-
Onset symptoms		-	-
Tremor, *n* (%)	12 (44.4)		
Bradykinesia, *n* (%)	7 (25.9)		
Motor deficits, *n* (%)	5 (18.5)		
Others, *n* (%)	3 (11.1)		
LEDD, mean (SD)	209.5 (128.6)	-	-
Education in years, median (IQR)	13 (8–17)	-	-

*Mean and SD were reported in case of normal distribution, while median and IQR were reported in case of non-normal distribution*.

*BMI, body mass index; HC, healthy controls; HandY, Modified Hoehn and Yahr; IQR, interquartile range; LEDD, Levodopa daily dose equivalent; MDS-UPDRS, Movement Disorder Society-sponsored revision of the Unified Parkinson's Disease Rating Scale; n, number; pwPD, persons with Parkinson's disease; SD, standard deviation; yrs-years*.

Chi-squared test

a and independent samples Student's t-test

b *were used to evaluate differences between pwPD and HC groups, as appropriate. P-values lower than 0.05 were considered significant*.

**Table 2:** Neuropsychological test scores of the pwPD group.

	**pwPD**	**Cut-off defining**
	**(n = 27)**	**pathological condition**
MoCA, mean (SD)	24.2 (2.9)	15.5 (≤)
TMT part A, median (IQR)	42 (30–56)	94 (≥)
TMT part B, median (IQR)	73 (58–139)	283 (≥)
TMT part B-A, median (IQR)	40 (18–68)	187 (≥)
Phonemic fluency, mean (SD)	35.4 (9.5)	17.35 (≤)
Semantic fluency, mean (SD)	42.9 (8.5)	25 (≤)

*Reported scores have been adjusted for age and education. Mean and SD were reported in case of normal distribution, while median and IQR were reported in case of non-normal distribution. The respective pathological cut-off scores are also shown*.

*IQR, interquartile range; MoCA, Montreal Cognitive Assessment; n, number; pwPD, persons with Parkinson's disease; SD, standard deviation; TMT, Trail Making Test; yrs-years*.

*MoCA scores were adjusted according to Santangelo et al. (24), TMT according to Giovagnoli et al. (28), phonological fluency according to Carlesimo et al. (27) and semantic fluency according to Novelli (26). The respective cut-off scores for pathological condition are reported*.

### CBF and GM Volume Comparison Between pwPD and HC

**Figure 1:**
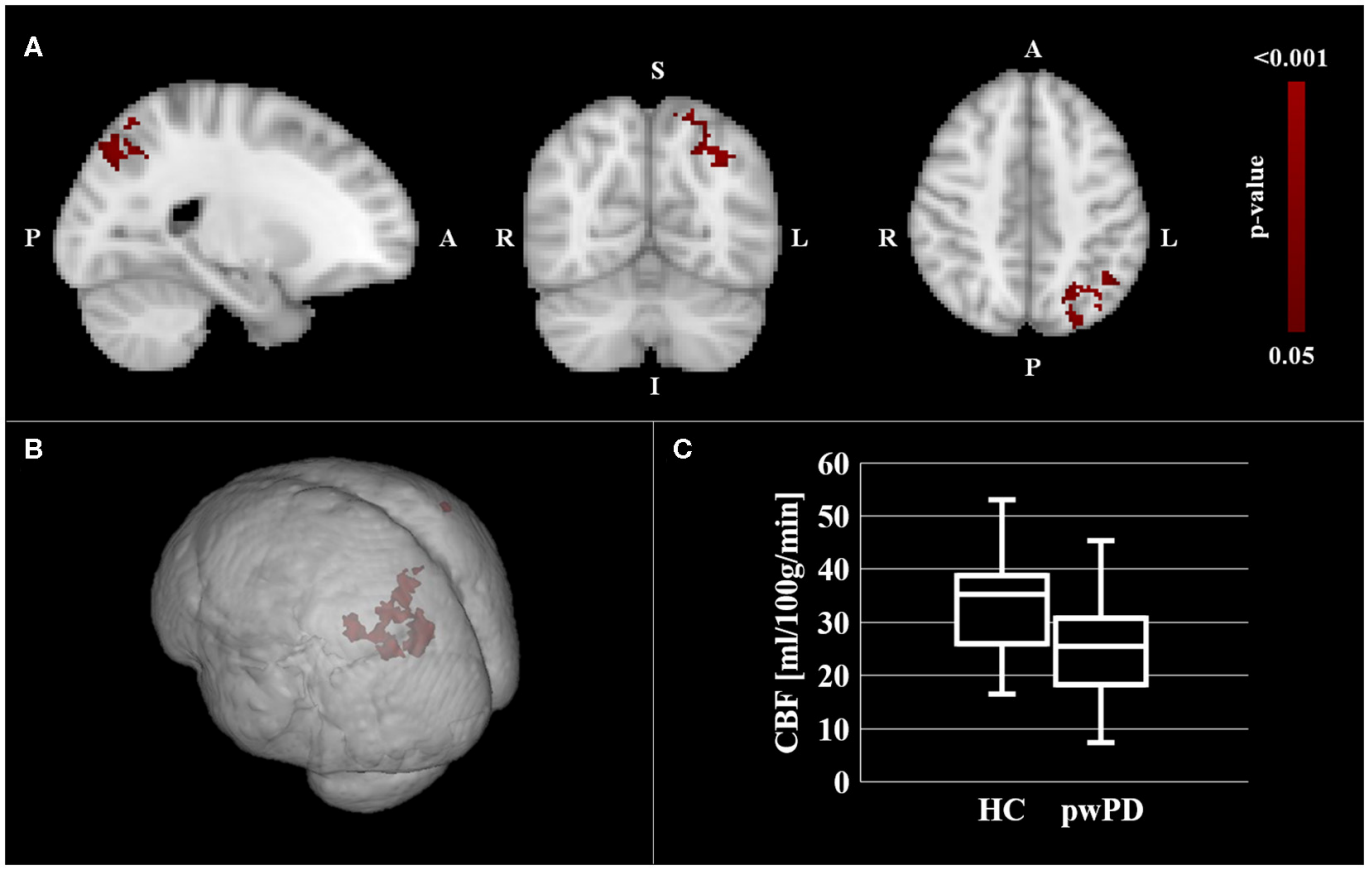
CBF comparison between pwPD and HC. The areas showing significantly lower CBF (p_FWE_ < 0.05) in pwPD with respect to HC are represented in MNI standard space (in red, **A,B**). The comparison of mean CBF within these areas between pwPD and HC is shown in **(C)**. CBF, cerebral blood flow; FEW, family wise error; HC, healthy controls; pwPD, people with Parkinson's disease.

Significantly lower CBF in pwPD with respect to HC was found within the SPL bilaterally and in the left IPL (Figure 1, in red). Specifically, hypoperfusion was present within the following clusters defined in the Mars's parietal cortex atlas: (41) left SPL C (16 mm^3^, p_peak_ = 0.049), right SPL C (64 mm^3^, p_peak_ = 0.049), left SPL D (896 mm^3^, p_peak_ = 0.037), left SPL E (936 mm^3^, p_peak_ = 0.038), left IPL D (552 mm^3^, p_peak_ = 0.037) and left IPL E (816 mm^3^, p_peak_ = 0.037) (Table 3).

**Table 3:** Classification of areas of significant hypoperfusion in pwPD group with respect to HC according to Mars' parietal atlas (41) and Sallet's dorsal-frontal atlas (40).

	**ROI**	**Associated functions**	**Left mm^**3**^ (p_**peak**_)**	**Right mm^**3**^ (p_**peak**_)**
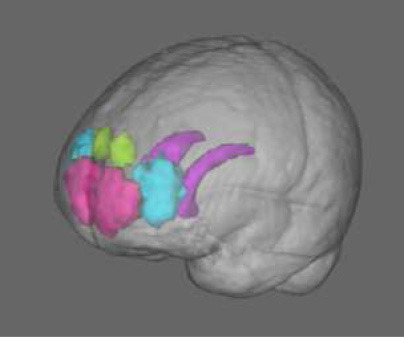	CN	Involved in procedural and associative learning, inhibitory control	-	-
	BA 9	Involved in attention, working memory and motor planning	-	-
	BA 10	Involved in working memory and multiple-task coordination	-	-
	BA 46	Involved in attention and working memory	-	-
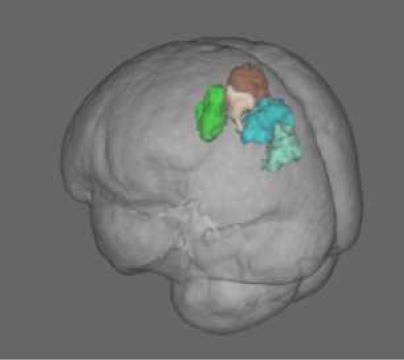	SPL A	Involved in motion processing using visual, tactile and auditory stimuli	-	-
	SPL B	Involved in reaching movements and adjustments when following a moving target	-	-
	SPL C	Involved in visually guided hand movements and adjustments when intentions are updated	16 (0.049)	64 (0.049)
	SPL D	Involved in mechanisms of attention or task-switching related to the execution of manual responses	896 (0.037)	-
	SPL E	Involved in the allocation of visual attention and in memorizing targets for intended eye movements	936 (0.038)	-
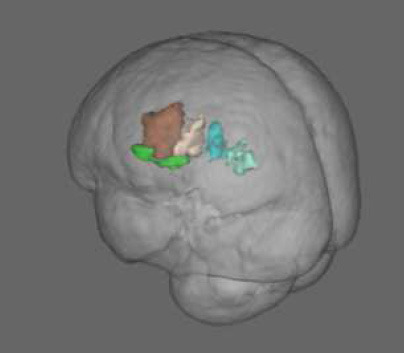	IPL A	Involved in tool use	-	-
	IPL B	Involved in object manipulation	-	-
	IPL C	Involved in exploratory decisions and changes of response strategy	-	-
	IPL D	Involved in reorienting of attention and reorienting of saccades (anterior part of the angular gyrus)	552 (0.037)	-
	IPL E	Involved in recognition memory (posterior part of the angular gyrus)	816 (0.037)	-

*The cluster dimension in mm^3^ and the most significant FWE-corrected p-value within the cluster (p_peak_) are reported*.

*BA, Brodmann area; CN, Caudate Nucleus; FWE, family wise error; HC, healthy controls; IPL, inferior parietal lobule; pwPD, people with Parkinson's disease; ROI, region of interest; SPL, superior parietal lobule*.

No significant GM volume differences were detected between pwPD and HC.

### Association With Neuropsychological Test Scores

**Figure 2:**
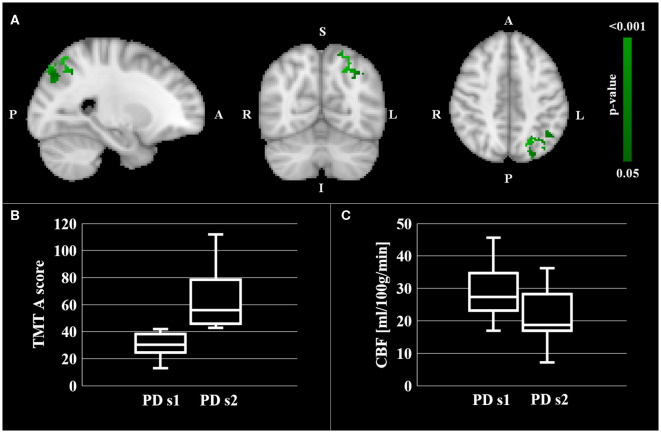
CBF comparison between pwPD with a TMT A score below the median TMT A value (PD s1 subgroup) and pwPD with a TMT A score over the median TMT A value (PD s2 subgroup). Areas showing significantly lower CBF (p_FWE_ < 0.05) in PD s2 subgroup are represented in MNI standard space (in green, **A**). The comparison of TMT A scores and mean CBF within these areas between the two pwPD subgroups are shown in the boxblots in **(B,C)**, respectively.

No significant correlation between the local CBF and the neuropsychological test scores was found within the regions of hypoperfusion. Significantly lower CBF in the left SPL and IPL was found for the subgroup of pwPD who performed poorer on TMT part A (i.e., higher TMT A score) with respect to the pwPD subgroup that performed better (Figure 2). Specifically, according to the Mars' parietal cortex atlas (41), the areas of significant difference were located within left SPL C (8 mm^3^, p_peak_ = 0.049), left SPL D (808 mm^3^, p_peak_ = 0.037), left SPL E (912 mm^3^, p_peak_ = 0.038), left IPL D (544 mm^3^, p_peak_ = 0.037) and left IPL E (640 mm^3^, p_peak_ = 0.035). The two pwPD subgroups obtained from the median split on TMT A score were matched for age (*p* = 0.239), sex (*p* = 0.546), H&Y (*p* = 0.981), MDS-UPDRS III (*p* = 0.720), LEDD (*p* = 0.519), MoCA (*p* = 0.720), phonemic fluency (*p* = 0.259), semantic fluency (*p* = 0.583) while presenting significant differences in terms of TMT B score (*p* = 0.006, with the subgroup performing poorer on TMT A performing poorer also to TMT B).

No significant CBF differences were observed when splitting the recruited pwPD group into subgroups according to the other neuropsychological test scores.

## Discussion

In the current study, CBF and GM volume were assessed with a voxel-wise approach in a group of early PD patients without dementia, focusing on the brain regions belonging to the visuospatial and executive systems, that are known to be affected in PD. The presence of parietal hypoperfusion in absence of parietal GM atrophy and its association with the TMT A score are the main findings that were observed.

Although the majority of PD studies that have previously detected parietal hypoperfusion or hypometabolism included cognitively impaired cohort of subjects (11, 15, 43–45), few works reported alterations in SPL (14, 16) and IPL (14, 46) even in absence of frank cognitive impairment. Cortical hypometabolism was detected in the posterior parietal cortex of non-demented PD patients with both phosphorus-31 magnetic resonance spectroscopy and FDG-PET (14). More recently Gonzalez-Redondo and colleagues showed small cortical areas of hypometabolism, including the angular gyrus, in cognitively normal pwPD (46). This result is in agreement with the hypoperfusion that we observed in left IPL D and IPL E in our pwPD cohort. The same PET-based study assessed brain metabolism also in MCI-pwPD and demented pwPD, and revealed hypometabolism in more extended brain areas in these two groups of subjects in comparison with the group of cognitively normal PD patients (46). Declining metabolic activity in the parietal cortex was further reported in a 2-year longitudinal PET study assessing an early stage PD cohort of subjects (12), indicating that reduced metabolism is a relatively early PD feature that gradually evolves as the disease progresses. Despite the several PET-based evidences of parietal hypometabolism in PD, ASL failed in consistently showing parietal hypoperfusion in early PD as of yet. However, CBF patterns were proposed as an accessible method to characterize and track progression of both motor and cognitive status in PD (17). Parietal perfusion changes may be subtle at the early stage of the disease, when dementia is not commonly established yet. However, focusing the analysis on specific ROIs that are known to be involved in cognitive processes typically altered in PD may increase the statistical power and may help to detect early perfusion changes occurring in pwPD in these areas. Due to the tight coupling of perfusion and metabolism in the brain, ASL has already been proposed as a non-invasive alternative to FDG-PET to assess metabolic abnormalities during cognitive decline in Alzheimer's disease (19). Our results support the assumption that this MRI technique might be useful to investigate the signature of cognitive impairment even in PD (17).

Interestingly, perfusion changes that were found in the pwPD enrolled in this study were not owing to parietal GM volume loss. This suggests that hypoperfusion in these regions may precede atrophy and exacerbate the neurodegenerative process by promoting oxidative stress and neuronal energy crisis (47). A previous cross-sectional study investigating both metabolism and atrophy in cognitively impaired and cognitively preserved pwPD concluded that hypometabolism may precede GM volume loss and may be progressively replaced by atrophy during cognitive worsening (46). The greater ability of FDG-PET to reveal significant PD-related alterations compared to MRI-VBM is in line with this hypothesis (45). Parietal GM atrophy may be a feature that is mainly associated with late stage of PD, when cognitive impairment is already established. Since functional changes seem to be already present (48) and more relevant than cortical atrophy in early PD (49), GM volume in these ROIs might not be the best option as a prodromal marker for PD itself and for the risk of developing PD-related dementia.

In the current study perfusion and GM volume were investigated specifically in the fronto-parietal circuit, known to be associated with cognitive impairment in PD (11). The prefrontal cortex is critical for many high-level cognitive abilities, including executive functions, while the parietal cortex is involved in lower level functions. Specifically, the SPL is commonly activated by visuomotor tasks requiring shifting spatial attention, engaging spatial working memory, reaching a visual target or making saccadic eye movements (50), whereas the IPL is critical for stimulus-driven reorienting of attention, self-perception, introspection and memory (51). In this framework, the here reported association between the TMT A score and CBF in some regions of the SPL and IPL of pwPD is noteworthy. Although no significant correlation was observed between the TMT A score and CBF locally, the subgroup of pwPD that performed poorer on TMT A showed lower CBF compared to the other subgroup. A similar finding was reported in a SPECT-based perfusion study in Alzheimer's disease (52). Specifically, lower SPL perfusion levels were found in Alzheimer's disease patients with poorer performance on TMT A in comparison with the subgroup performing better on the test (52), suggesting that functional activity in SPL is highly associated with performance at TMT A. TMT A primarily tests visual-perceptive skills and graphomotor speed. It is thus not surprising that the main SPL and IPL clusters showing lower CBF associated with poorer TMT A performance are specifically involved in allocation of visual attention, reorienting of saccades, memorizing targets for intended eye movements and attention-switching related to the execution of manual responses. As lower parietal CBF was associated with poorer TMT-A performance, parietal CBF may be an early MRI marker for cognitive decline in PD.

Neither GM volume nor perfusion alterations were observed in the caudate nucleus and in the prefrontal cortex of the enrolled group of pwPD. Parietal CBF alterations may precede gross caudate denervation and changes in the prefrontal cortex, and may be an earlier predictor of cognitive decline. Although PD pathophysiology is characterized by striatal dopaminergic denervation, caudate dysfunction is generally observed in pwPD at advanced stages (53). In an FDG-PET study comparing brain metabolism of cognitively preserved pwPD, MCI-pwPD and demented pwPD with HC, caudate hypometabolism was observed only in the group of demented pwPD (46), indicating that metabolic changes in this region unlikely occur prior to the onset of cognition-related functional decline. Similar considerations can be made about our findings within the prefrontal ROIs. Frontal hypoperfusion and hypometabolism were observed in pwPD presenting with established dementia. GM atrophy in these areas was also reported (54, 55). Our findings of no perfusion and GM changes in the caudate and in the prefrontal ROIs could suggest that the dopaminergic pathways that project from the substantia nigra into BA 9, 10, and 46, passing through the caudate, may be still relatively spared in our patient sample.

The main limitation of this study is the relatively limited neuropsychological evaluation of pwPD. Assessing the cognitive performance with scales that are validated for use in PD (56) is warranted to detect possible MCI and to clarify the role of CBF as a predictor of cognitive decline in pwPD. Furthermore, weaknesses of the study include the relatively limited sample size and the heterogeneity of the pwPD group. Different clinical phenotype and laterality onset were suggested to influence the disease severity and its patterns of progression (57, 58). Therefore, these aspects should be considered as confounding factors in future analysis performed in larger groups of subjects. Finally, this study is cross-sectional in nature. A longitudinal study would allow to better understand the link between perfusion alterations and cognitive decline. In addition, extending the analysis to other subcortical brain areas that are known to be associated with PD (e.g., putamen, globus pallidus, thalamus) is warranted.

In conclusion, our findings suggest that ASL MRI may be sensitive to parietal changes associated with cognitive decline in PD, even in absence of concurrent established GM atrophy. Reduced perfusion in the parietal cortex may be associated with the risk of developing cognitive impairment, but longitudinal studies in a larger cohort of pwPD are needed to confirm this hypothesis. Knowledge of the risk of cognitive decline, particularly at early phases of the disease, is crucial to define prompt pharmacological and rehabilitation treatments. Since engaging in physical activity and cognitive activities were proposed as strategies to attenuate neurodegeneration by promoting neurovascular health and brain plasticity (59), investigating the impact of multi-factorial rehabilitation on parietal CBF in early pwPD would be a further interesting future development.

## Data Availability Statement

The datasets generated for this study are available on request to the corresponding author.

## Ethics Statement

The studies involving human participants were reviewed and approved by Fondazione Don Carlo Gnocchi Ethics Committee. The patients/participants provided their written informed consent to participate in this study.

## Author Contributions

LP, SD, ML, NB, AP, and FB contributed conception and design of the study. RN and MM recruited PD patients. ML and FR enrolled HC. FB and MM performed the clinical evaluation of PD patients. SD and FR performed the neuropsychological evaluation of PD patients. LP performed the image processing, the statistical analysis, and wrote the first draft of the manuscript. All authors contributed to manuscript revision, read, and approved the submitted version.

## Conflict of Interest

The authors declare that the research was conducted in the absence of any commercial or financial relationships that could be construed as a potential conflict of interest.
